# Emergence of enterovirus D68 clade D1, France, August to November 2018

**DOI:** 10.2807/1560-7917.ES.2019.24.3.1800699

**Published:** 2019-01-17

**Authors:** Antonin Bal, Marina Sabatier, Thierry Wirth, Marianne Coste-Burel, Mouna Lazrek, Karl Stefic, Karen Brengel-Pesce, Florence Morfin, Bruno Lina, Isabelle Schuffenecker, Laurence Josset

**Affiliations:** 1 Centre National de Référence des Enterovirus et Parechovirus, Hospices Civils de Lyon, Lyon, France; 2 Laboratoire de Virologie, Institut des Agents Infectieux, Hôpital de la Croix-Rousse, Hospices Civils de Lyon, Lyon, France; 3 Université Lyon 1, Faculté de Médecine Lyon Est, CIRI, Inserm U1111, CNRS UMR5308, Virpath, Lyon, France; 4 Laboratoire Commun de Recherche Hospices Civils de Lyon-bioMerieux, Centre Hospitalier Lyon Sud, Pierre-Bénite, France; 5 Laboratoire Biologie Intégrative des Populations, Evolution Moléculaire, EPHE, PSL Université, Paris, France; 6 Institut Systématique, Evolution, Biodiversité (ISYEB), EPHE, MNHN, CNRS, Sorbonne Université, Paris, France; 7 Laboratoire de Virologie, UIC9 CIC infectieux, Centre Hospitalier Universitaire de Nantes, Nantes, France; 8 Laboratoire de Virologie, EA3610, Centre Hospitalier Universitaire de Lille, Université de Lille, Lille, France; 9 INSERM U1259, Université de Tours, Tours, France; 10 Laboratoire de Virologie and CNR VIH-Laboratoire Associé, Centre Hospitalier Régional Universitaire de Tours, Tours, France

**Keywords:** enterovirus D68, emergence, whole genome sequencing, metagenomics, respiratory infection, outbreak

## Abstract

We report a seasonal increase of enterovirus D68 (EV-D68) cases in France, with 54 cases detected between 19 August and 14 November 2018. Molecular typing revealed that 20 of 32 of the isolates belonged to clade D1, only sporadically detected before in France. Median age of D1-cases was 42 years, 10 developed severe respiratory signs and one had neurological complications. The 2018-D1 viruses showed a genetic divergence of 3.34 % with D1 viruses identified previously.

Since August 2018, a seasonal wave of enterovirus D68 (EV-D68) cases has been reported in France. Here, we present the molecular and clinical characteristics of the EV-D68 cases with a special focus on D1-associated cases and on severe cases.

## Enterovirus D68 detection and typing

**Figure 1:**
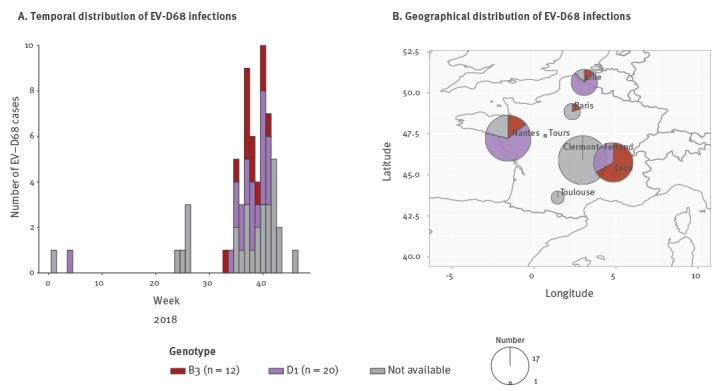
(A) Temporal and (B) spatial distribution of enterovirus D68 infections, France, 2018 (n = 61)

A total of 61 EV-D68 infections were reported in France from 1 January–18 December 2018, as part of the enterovirus infections national surveillance that comprises of a network of 35 hospital laboratories (Figure 1). Systematic screening for EV-D68 of all enterovirus/rhinovirus-positive respiratory samples was performed in three university hospitals (Lyon, Nantes, and Clermont-Ferrand). In other hospital laboratories, EV-D68 testing was performed occasionally for cases presenting with severe respiratory or neurological illness. EV-D68 could also be identified through routine genotyping of EV-positive samples. 

EV-D68: enterovirus D68.

Panel B: Pie charts show the frequency of EV-D68 genotypes. The radius is proportional to number of cases.

Of 61 EV-D68 cases, 54 (88.5%) were detected from 19 August–14 November with a peak in cases (n = 10) in week 40 (15–21 October) (Figure 1A). We carried out molecular typing for 32 of 61 EV-D68-positive samples by Sanger sequencing of the complete viral protein 1 (VP1) sequence (n = 25), partial VP1 sequence (n =6), or VP4-VP2 sequences (n = 1) as previously described [1,2]. Phylogenetic analysis found that the majority of EV-D68 isolates belonged to clade D1 (20/32), while the remaining belonged to clade B3 (12/32). Co-circulation of D1- and B3-viruses was detected throughout the 2018 epidemic (Figure 1A) and all over France (Figure 1B).

## Clinical characteristics of enterovirus D68 clade D1- or B3 -positive patients

Among the 20 D1-positive cases (median age: 42 years; range: 0.1–79 years), the majority of infections (n = 14) were diagnosed in adults, with only six cases identified in children aged 5 years or below. Ten patients presented with severe respiratory signs including severe asthma (n = 2), acute respiratory distress (n = 4) and acute cardiorespiratory decompensation (n = 4). Among these severe respiratory cases, nine had underlying co-morbidities (Table). Two patients in their early 70s with underlying malignancies died from cardio-respiratory failure.

**Table:** Demographic and clinical characteristics of enterovirus D68 clade D1- or B3- positive patients, France, 2018 (n = 62)

Clinical features	Number of D1-cases (n = 20)	Number of B3-cases (n = 12)
**Sex**		
Male	10	7
Female	10	5
**Age group (years)**		
< 5	6	7
5–17	1	3
18–64	8	1
> 65	5	1
**Underlying co-morbidities**		
Haematological malignancies^a^	7	3
Carcinoma	1	0
Solid organ transplantation	1	1
Obesity (BMI > 30 kg/m^2^)	1	0
Pulmonary disease	2	3
Cardiovascular disease	1	0
Congenital disease	1	1
**Total**	**14**	**8**
**Severe respiratory symptoms**		
Severe asthma	2	2
Acute respiratory distress	4^b^	3
Acute cardiorespiratory decompensation	4^c^	0
**Total**	**10**	**5**
**Neurological symptoms**	1	1
**Deaths**	2	0

BMI: body mass index.

^a^ Haematological malignancies includes: lymphoma, acute leukaemia, multiple myeloma and essential thrombocythaemia.

^b^ One case association with a *S.pneumoniae* respiratory infection and a myopericarditis.

^c^ One case association with acute pancreatitis.

One patient in their late 60s developed neurological signs shortly after influenza-like prodromal symptoms. They presented with an acute aphasia associated with upper and lower right limb weakness and facial paralysis, compatible with cranial nerve impairment. Cerebrospinal fluid analysis was compatible with aseptic meningitis (white blood cells (WBC): 34 per mm^3^; Norm: five or less WBC per mm^3^; 95% of lymphocytes) but extensive microbiological investigations, including enterovirus PCR testing, were negative. Neurological symptoms improved within 10 days. Brain MRI did not show rhombencephalitis. No medullar MRI was performed. 

Of the 12 B3-associated cases the median age was 4.5 years (range: 0.2–65 years). The clinical spectrum was dominated by respiratory symptoms primarily occurring in children aged 5 years or below (n = 7). Eight B3-associated cases had an underlying disease and six cases developed a severe infection, including one case of rhombencephalitis in a child aged 5 years (Table).

## Molecular investigations

**Figure 2:**
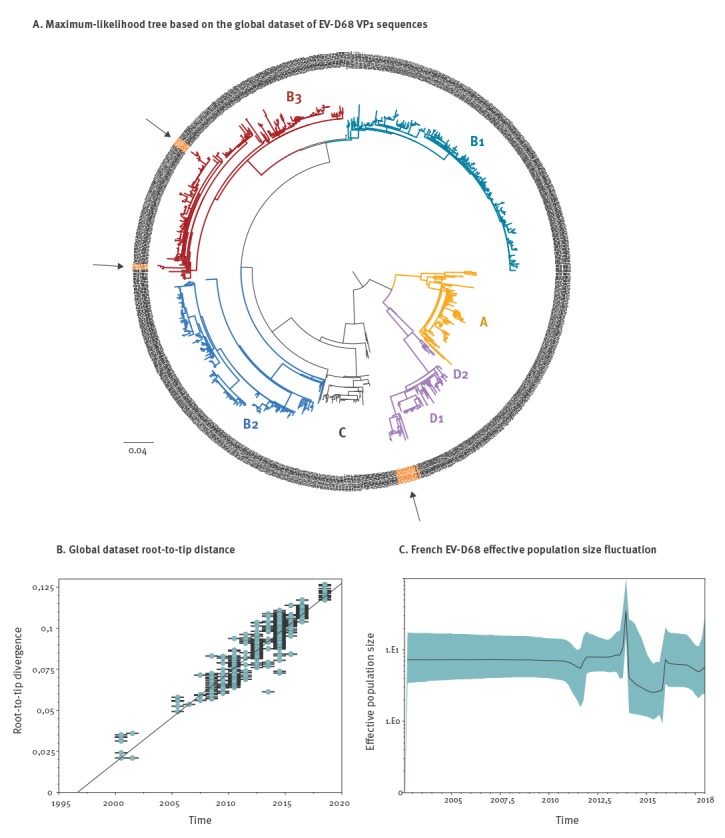
Evolutionary history of enterovirus D68, 1962–2018

To characterise the global dynamics of EV-D68 clade D1 strains over time, we performed a phylogenetic analysis of the full VP1 sequences generated in this study from patients from three different administrative regions of France (GenBank accession numbers: MK121710-MK121730) in comparison to all published worldwide VP1 sequences (global dataset: 810 sequences available in GenBank at 11 Oct 2018) [3] (Figure 2A). This dataset showed a strong temporal signal (Figure 2B) and the time to most recent common ancestor (TMRCA) of clade D1 was estimated to date back to 2010 (95% highest posterior density, 2008–2011) according to Bayesian inference. The first clade D1 viruses were identified in 2011 (East Asia), but since then and until 2018, they have been rarely detected (Asia, 2011-2016, 33 sequences (Hong-Kong: n=14, China: n=12, Taiwan: n=5, Japan: n=2); North America, 2013-2014, 4 sequences (Canada: n=2; US: n=2); Europe, 2012-2014, 34 sequences (Germany: n=20, France: n=9, Italy: n=1, Spain: n=2, Denmark: n=2) based on all whole genome sequences (n=508) and VP1 sequences (n=810) available in GenBank at 11 Oct 2018) . 

EV-D68: enterovirus D68.

Panel A: Maximum-likelihood tree of the global dataset of EV-D68 VP1 sequences based on the general-time-reversible (GTR) model of DNA sequence evolution. The arrows indicate strains involved in the 2018 outbreak (highlighted in orange). The scale shows the length of branch representing 0.04 nt substitutions per site.

Panel B: Root-to-tip genetic distances are plotted against sampling dates. The figure illustrates a positive correlation of divergence with sampling date, and, hence, a significant increase of nt sequence variation over the sampling time interval.

Panel C: Bayesian skyline plot showing the effective population size fluctuations of EV-D68 in the French cohort. The black line represents the median estimate of the estimated effective population size. The shade represents the upper and the lower estimates of 95% confidence interval.

In France, clade B2 and B3 largely predominated in 2014 and 2016, while clade D1 viruses were detected for the first time in 2012 (one isolate) and represented only 9 of 201 (4.5%) of the EV-D68 detected in France in 2014. The 2018 D1 isolates showed a nt divergence in the VP1 gene of 3.34% compared with the D1 strains previously characterised (Figure 2A).

According to the Bayesian skyline analysis, the 2018 outbreak remains relatively modest (within the limits of sequencing data publicly available) and has not reached the levels of the 2014 or 2016 episodes (Figure 2C). In addition to conventional molecular typing, the whole genome of thirteen EV-D68 were sequenced using a validated metagenomic next-generation sequencing assay [4], including D1 strains from 2012 (n = 1), 2014 (n = 4) and 2018 (n = 2), as well as B3 isolates from 2016 (n = 3) and 2018 (n = 3) (GenBank accession numbers: MK105976- MK105989). No evidence of inter-clade recombination event involving the clade D1 was found using standard recombination detection package [5].

## Discussion and conclusion

Since the global emergence of EV-D68 in 2014, this virus has been associated with a wide range of infections including neurological and severe respiratory diseases [6]. As EV-D68 circulation is driven by a biennial pattern in European countries, an upsurge of EV-D68 cases was expected in 2018 [3]. An increase of EV-D68 detection was detected in France during autumn 2018 and was recently reported in Wales [7]. Detection of EV-D68 was reported in other European countries as well (Sofie Midgley, European Centre for Disease Prevention and Control National Microbiology Focal Points meeting, October 2018). The burden and circulation of EV-D68 in France in 2018 is likely under-represented in this study, as cases were identified through hospital-based surveillance and only 3/35 laboratories performed systematic screening of EV-D68.

We found that EV-D68 clade D1 was the predominant circulating genotype, partially replacing the B3 genotype that was predominant in 2016 [3]. Viruses from clade D1 were initially detected in East Asia in 2011 but since then, only a few detections have been reported worldwide [8]. A continuous emergence and replacement of EV-D68 clades have been observed since 2010, in particular, a low circulation of the B3 lineage was noticed before its global spread in 2016 [3,9-11]. We also found that D1 viruses were mainly detected in adult cases, while B3 viruses were mainly detected in children. This age-effect depending on the EV-D68 circulating genotype had already been observed in previous studies, which found that viruses from clade D1 (previously classified as clade A2) predominantly infected adults [1,12].

In addition to severe respiratory illness, neurological complications were observed in one patient with a D1 EV-D68 infection, suggesting a neurotropic potential of this genotype. EV-D68 neurotropism was recently demonstrated in a neuroblastoma-derived neuronal cell line model, indicating that only contemporary strains of EV-D68 (including US/KY/14–18953 strain belonging to the clade D1) have acquired neurovirulence over time [13]. EV-D68 neurotropism could partly explain the spike of acute flaccid myelitis cases observed in the summer and early Autumn of 2014, 2016 and 2018 in the US [10,14]. Our data confirm that EV-D68 can be associated with severe respiratory disease in immunocompromised individuals particularly in patients with haematological malignancies [15].

Finally, using a metagenomic approach we provide the first European full-length genomes of clade D1 viruses, which contributes to improve our knowledge regarding EV-D68 genetic diversity. Whole genome sequence analysis did not find evidence of interclade recombination events [8,16] that could have led to the emergence of the D1 genotype.

The data presented here underline the value of extensive molecular and clinical investigations to fully describe the complex spectrum of EV-D68 infections. A global real-time surveillance of EV-D68 should be maintained and emphasised to rapidly alert epidemiologists, microbiologists and clinicians.
